# Polarization-aware UAV deployment for reliable MIMO communication in forested environments

**DOI:** 10.1371/journal.pone.0343057

**Published:** 2026-02-26

**Authors:** Mohamed Shalaby

**Affiliations:** Electrical Engineering Department, Imam Mohammad Ibn Saud Islamic University (IMSIU), Riyadh, Saudi Arabia; Beijing Technology and Business University, CHINA

## Abstract

Unmanned Aerial Vehicles (UAVs) equipped with Multiple-Input Multiple-Output (MIMO) communication systems are increasingly deployed to restore or extend connectivity in forested and remote regions where terrestrial infrastructure is unavailable. However, radio propagation through vegetation is strongly affected by polarization-dependent scattering, attenuation, and depolarization, which can severely degrade link reliability. This study investigates polarization-aware UAV deployment as a means to enhance air-to-ground communication performance under dense canopy conditions. A vegetation-aware propagation model is developed using the Debye relaxation framework combined with Kramers–Kronig relations to capture the dielectric response of moist foliage. Cross-Polarization Discrimination (XPD) is identified as a dominant factor influencing signal quality, exhibiting non-monotonic variations that complicate UAV positioning. To address this, the Crow Search Algorithm (CSA) is employed to determine optimal UAV locations that minimize XPD between orthogonal polarization channels. Simulation results demonstrate that polarization-aware optimization significantly improves link robustness compared to traditional path-loss-based strategies, particularly at higher frequencies. The findings highlight the importance of integrating polarization awareness into UAV communication planning for critical missions such as search-and-rescue and post-disaster recovery in vegetated environments.

## Introduction

UAVs have become essential components of modern wireless communication networks due to their flexibility, mobility, and ability to provide rapid connectivity. These features are particularly valuable in scenarios where ground infrastructure is limited or damaged, such as natural disasters, remote terrains, and rescue missions in forested regions. When integrated with Multiple-Input Multiple-Output (MIMO) systems, UAVs can deliver high-capacity and resilient links through spatial diversity and beamforming capabilities.

However, establishing reliable UAV-ground communication in vegetated environments remains a critical challenge. Dense foliage introduces complex propagation impairments, including scattering, absorption, depolarization, and cross-polarization coupling. These effects are frequency-dependent and governed by the dielectric properties of vegetation, which vary significantly with moisture content, temperature, and biological structure. Among these impairments, XPD has emerged as a key limiting factor in maintaining MIMO channel orthogonality—especially at higher frequency bands used in 5G and 6G systems.

Conventional UAV placement strategies typically focus on minimizing path loss to improve signal strength [1,2]. However, our investigation reveals that XPD does not behave monotonically with respect to UAV position, and several local minima exist. In addition, XPD can reach values that rende MIMO communication links completely non-operational. Therefore, optimizing for XPD, rather than path loss alone, is essential to ensure communication robustness in such environments.

Despite the growing body of research on vegetation-induced attenuation and UAV-based communication, there remains a gap in polarization-aware UAV placement strategies that explicitly account for the spatial behavior of XPD over forested terrain.

This work addresses that gap by modeling polarization-dependent signal degradation using realistic dielectric models of vegetation and quantifying the influence of XPD over a wide frequency range. We demonstrate that XPD, rather than path loss alone, serves as the dominant factor in determining optimal UAV positions. To tackle the non-monotonic nature of XPD and the presence of multiple local minima, we apply the CSA algorithm to globally optimize UAV deployment. Our findings show that minimizing XPD results in significantly more robust MIMO communication performance in forested environments—especially for rescue operations where reliable links are critical.

## Literature review

Radio wave propagation in forested environments is subject to complex interactions influenced by vegetation structure, dielectric properties, moisture content, and wave polarization. Understanding these interactions is critical for establishing robust wireless communication links, especially for UAV-ground channels in rescue and disaster scenarios.

Empirical and semi-empirical models have been widely used to quantify vegetation-induced attenuation. [3,4] reviewed traditional models and highlighted the role of environmental factors such as wind and rainfall, as well as antenna height. Similarly, [5] and [6] statistically modeled vegetation-related fade margins, showing that link budgets can be improved by incorporating vegetation parameters. [7] further demonstrated the dominance of lateral waves above 200 MHz, emphasizing the importance of horizontal propagation analysis in forested terrains. Other early foundational models such as those by [8] and [9,10] remain influential in understanding microwave transmission through vegetated canopies.

Vegetation’s electromagnetic behavior is closely tied to its dielectric properties, which vary significantly with frequency, moisture content, and species-specific morphology. [11] provided measurements of dielectric constants for wood samples at microwave frequencies, showing that anisotropy and water content are key factors. [12] extended this to tropical hardwoods, which are highly relevant in many equatorial regions. These findings support the use of frequency-dependent dielectric models—such as the Debye relaxation model—for accurate characterization of foliage and wood, particularly in moist environments. Further supporting this, work by [13,14], and [15] highlighted coherent and morphological impacts on propagation, while [16] used SAR observations to confirm a correlation between vegetation density and signal attenuation. See also [10,17–22] for related analyses in urban, IoT, and diverse vegetative contexts.

Polarization effects are another critical concern in vegetated environments. Studies have shown that wave depolarization and cross-polarization coupling significantly degrade signal quality. [23] and [24] found that vertically polarized waves suffer higher attenuation—up to 15 dB more than horizontally polarized waves—due to foliage structure. [25] observed differential polarization behavior in dense canopies, further corroborating the need for polarization-specific modeling. These observations are reinforced by the theoretical reviews and the modeling work of [26], which provide comprehensive descriptions of polarization behavior through vegetated media. More recent measurement-based studies at mmWave frequencies, such as those by [27,28], and [29], confirmed that polarization discrimination worsens significantly with frequency and vegetation density, making XPD a limiting factor in MIMO system performance.

Efforts to integrate UAVs into vegetated communication environments have largely focused on minimizing path loss. [29] and [30] modeled mmWave propagation in vegetated and suburban areas, incorporating angular dependencies and correction factors to better predict attenuation. While these approaches provide improvements over earlier models, they typically neglect polarization dynamics and do not address the spatial non-uniformity of XPD. Other works, such as [31], have addressed V2V and IoT communication in vegetated zones but similarly lacked a focus on polarization-aware UAV positioning. Additionally, several measurement studies have explored vegetation impact in more specialized environments such as mango greenhouses and agricultural fields [32], providing diverse empirical baselines. Similar field studies by [33], and [34] further enrich our understanding of wave behavior in structured natural environments.

While these prior studies provide valuable insight into attenuation mechanisms and wave-vegetation interaction, they fall short in addressing the combined effects of cross-polarization interference and UAV spatial optimization. Specifically, the non-monotonic spatial behavior of XPD has not been analyzed, and no optimization framework has been proposed to globally minimize XPD through UAV repositioning. In this study, the UAV employs two patch antennas oriented at $\;+45^{\circ }$ and $-45^{\circ }$ relative to the vertical axis, mounted on a tilted plane with respect to the vertical plane. This configuration ensures orthogonal polarization states, which form the basis for the subsequent XPD analysis. This analysis addresses that gap by (i) developing a frequency-dependent dielectric model for moist vegetation using Debye and Kramers-Kronig formulations, (ii) quantifying XPD variation with UAV location and frequency, and (iii) applying the Crow Search Algorithm to identify UAV positions that minimize XPD and enhance MIMO communication quality for rescue teams operating in forested environments.

## Vegetation attenuation model

The excess attenuation *A*, due to the presence of vegetation, is given by:

$$A=A_{m}\left(1-e^{-\frac{\gamma d}{A_{m}}}\right)$$ (1)

where:

- *d*: length of path within woodland (m)
- *γ*: specific attenuation for very short vegetative paths (dB/m)
- *A*_*m*_: maximum attenuation for one terminal within a specific vegetation type (dB)

Curve fitting of specific attenuation as a function of frequency *f* for both polarizations is given by:

$$\gamma_{V}=10^{-8}\cdot f^{0.8231}$$ (2)

$$\gamma_{H}=2\cdot 10^{-10}\cdot f^{1.0014}$$ (3)

where *f* is in Hz and *γ* is in dB/m.

$$A_{m}=A_{1}\cdot f^{\alpha }$$ (4)

Coefficients *A*_1_ and *α* depend on the type of vegetation. Values of these parameters are shown in Table 1 for some types of forest [35].

**Table 1:** Vegetation attenuation coefficients [35].

Vegetation Type	Freq. Range (MHz)	*A*_1_	*α*
Tropical Trees (Brazil)	900–1800	0.18	0.752
Various Trees (France)	900–2200	1.15	0.43
Mixed Forest (Russia)	105.9–2117.5	1.37	0.42

It is important to note that these attenuation models are not purely analytical but are derived from extensive experimental measurements conducted and standardized by the ITU-R (Recommendation P.833-8)[35]. Decades of empirical campaigns across different vegetation types and frequency bands underpin these models, which ensures that the dielectric constants and attenuation coefficients used in this work rest on a solid experimental foundation. In this sense, our analysis is not a purely theoretical or simulation-only study; rather, it extends these validated experimental datasets into the context of polarization-aware UAV deployment.

These equations show that attenuation increases with frequency and that horizontally polarized waves experience different loss than vertically polarized waves. This disparity in attenuation between the two polarizations when propagating through vegetated environments is a primary contributor to cross-polarization discrimination (XPD), as will be further discussed.

## Analysis of vegetation loss main constituents

Vegetation loss is attributed to trees leaves and branches and the water content in these elements. If the dielectric constant for leaves is unknown, it can be computed in the following way [35],

$$\varepsilon_{l}=3.1686+\frac{28.938}{1+j\frac{f}{18}}-j\frac{0.5672}{f}=\varepsilon_{l}^{\prime }+j\varepsilon_{l}^{\prime \prime }$$ (5)

$$\varepsilon_{l}^{\prime }=3.1686+\frac{28.938}{1-{\left(\frac{f}{18}\right)}^{2}}$$ (6)

$$\varepsilon_{l}^{\prime \prime }=-\frac{1.6077f}{1-{\left(\frac{f}{18}\right)}^{2}}-\frac{0.5672}{f}$$ (7)

**Fig 1:**
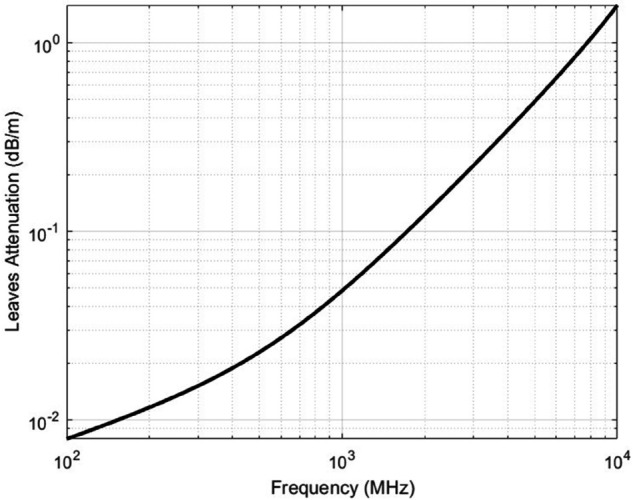
Attenuation caused by leaves across varying frequencies. This figure illustrates how dielectric properties of leaves, primarily influenced by moisture, result in frequency-dependent signal losses.

$\varepsilon_{l}$ is the dielectric constant of leaves, where $\varepsilon_{l}^{\prime }$ and $\varepsilon_{l}^{\prime \prime }$ are its real and imaginary parts. Fig 1 demonstrates the attenuation effects introduced by foliage leaves across different frequencies. As shown, the signal loss increases with frequency, highlighting the frequency-dependent nature of attenuation caused by the dielectric properties of leaves. These properties, largely governed by moisture content, significantly influence the degree of signal degradation, especially at higher frequencies commonly used in wireless communication systems. Although the analysis is limited to 10 GHz, Fig 1 suggests that these trends can be extrapolated to cover current 6G frequencies and future mmWave applications.

The second main constituent of foliage is trees branches. Loss introduced by trees branches is expressed as [35]

$$\varepsilon_{b}=\varepsilon_{b}^{\prime }\left(1+j\tan(\delta_{b})\right)$$ (8)

**Fig 2:**
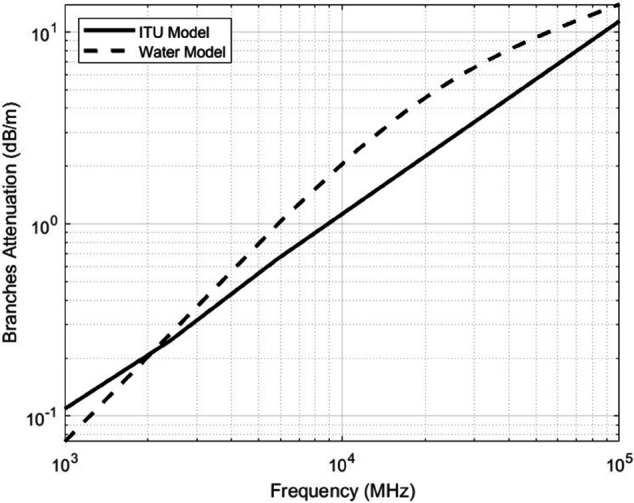
Electromagnetic loss induced by tree branches versus frequency. The attenuation trends are derived based on the water-like dielectric behavior of moist wood, emphasizing stronger losses at higher frequencies. The figure shows also branches attenuation according to the ITU model based on measurements [35].

$\varepsilon_{b}$ is the dielectric constant of branches, where $\varepsilon_{b}^{\prime }$, is the real part of the dielectric constant and $\tan(\delta_{b})$ is the loss tangent.

where $\varepsilon_{b}^{\prime }$ and $tan(\delta_{b})$ are computed at frequency f by linear interpolation of the values given in Table 2. Fig 2 shows the loss attributed by trees branches.

**Table 2:** Dielectric Constant and Loss Tangent of Wood (Moisture = 40%, Temp = 20^°^C) [35].

Frequency (GHz)	Dielectric Constant $\varepsilon_{b}^{\prime }$	Loss Tangent $\tan(\delta_{b})$
1	7.2	0.29
2.4	6.2	0.30
5.8	6.0	0.37
100	5.3	0.43

In analyzing the electromagnetic properties of tree branches, it is crucial to account for their dielectric characteristics, which significantly influence signal attenuation and scattering. The dielectric constant of wood is largely dependent on its moisture content, as water is the dominant contributor to its permittivity. Given that fresh wood can contain a substantial amount of water, particularly in living trees, its electromagnetic response is strongly influenced by the dielectric behavior of water. At microwave and millimeter-wave frequencies, the complex dielectric constant of water varies with frequency due to molecular relaxation effects. Since wood with high moisture content exhibits similar dielectric properties to water, it is a reasonable first approximation to model the dielectric constant of tree branches using the known frequency-dependent behavior of water. This assumption provides a practical way to estimate attenuation and propagation effects in forested environments, especially for remote sensing and communication applications. By profiting from established models for the dielectric properties of water, we can approximate the response of tree branches with significant moisture content, facilitating a more straightforward analysis of wave propagation in woodland environments.

To accurately model the dielectric constant of water, particularly in the microwave and millimeter-wave frequency ranges, we employed the well-established Debye relaxation model [36,37]. This model describes the frequency-dependent complex permittivity of polar liquids, such as water, and characterizes both the real part (dielectric constant) $\varepsilon^{\prime }$ and the imaginary part (dielectric loss) $\varepsilon^{\prime \prime }$ as functions of frequency.

$$\varepsilon^{\prime }(\omega )=\varepsilon_{\infty }+\frac{\varepsilon_{s}-\varepsilon_{\infty }}{1+(\omega \tau {)}^{2}}$$ (9)

$$\varepsilon^{\prime \prime }(\omega )=\frac{(\varepsilon_{s}-\varepsilon_{\infty })\omega \tau }{1+(\omega \tau {)}^{2}}+\frac{\sigma }{\omega \varepsilon_{0}}$$ (10)

where the parameters in these formulas and their approximate values for water at room temperature (around 25^*o*^*C*) are:

- $\epsilon_{s}$ is the static permittivity (low-frequency limit)  = 78.4,
- $\epsilon_{\infty }$ is the high-frequency permittivity limit  = 4.9,
- *τ* is the relaxation time $=8.27\cdot 10^{-12}s$,
- *ω* is the radian frequency,
- *σ* is the ionic conductivity ≈0 for pure water,
- $\epsilon_{0}$ is the permittivity of free space $=8.854\cdot 10^{-12}F/m$.

By utilizing this model, we determined the dielectric properties of water across a broad frequency spectrum. These values enabled the calculation of the frequency-dependent dielectric constant and dielectric loss within the microwave and millimeter-wave ranges. The resulting data provides a reference for estimating the dielectric behavior of moisture-rich wood, facilitating further analysis of wave propagation in forested environments. Fig 2 presents a comparison between branch-induced signal loss estimated using the ITU model and the loss calculated based on the water content of the branches. The comparison reveals that water content is the dominant factor contributing to signal attenuation, indicating that the majority of the loss attributed to branches can be primarily explained by their moisture levels.

These dielectric parameters form the basis for modeling vegetation-induced attenuation, which is essential in evaluating electromagnetic propagation in forested environments. The following section extends this by examining how these properties affect polarization behavior and XPD in real-world scenarios.

## XPD analysis

In UAV-based MIMO communication systems, maintaining signal integrity is crucial, especially when operating in complex propagation environments such as vegetated regions. A key challenge in these scenarios arises from the polarization-dependent phase shift caused by the differential propagation of horizontally and vertically polarized waves through vegetation. As electromagnetic waves traverse foliage, the real part of the dielectric constant $\left(\epsilon^{\prime }\right)$ varies slightly between the two polarizations. While this difference may seem small, it leads to distinct phase velocities for each polarization, causing a gradual accumulation of phase difference over distance. This effect becomes particularly pronounced at high frequencies (e.g., millimeter-wave or terahertz bands) due to the direct proportionality between phase shift and frequency. In a UAV-MIMO system, where polarization diversity is often employed to improve spectral efficiency and mitigate fading, this polarization-induced phase shift can lead to depolarization effects, and reduced MIMO channel orthogonality. This can significantly impact polarization diversity techniques used to enhance data rates and coverage. Moreover, as the UAV dynamically changes altitude, the angle-dependent interaction with vegetation further alters polarization-dependent losses, introducing additional signal impairments. Understanding and compensating for this effect is essential to ensure robust and high-performance MIMO communication for mobile users in forested and suburban environments.

**Fig 3:**
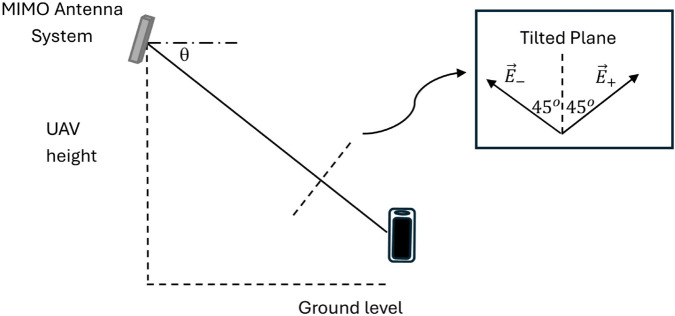
Configuration of the two patch antennas oriented at $\;+45^{\circ }$ and $-45^{\circ }$ with respect to the vertical axis, mounted on the tilted plane. This setup ensures orthogonal polarization states for the MIMO system, providing the basis for the analysis of polarization-specific losses and phase shift in vegetated channels.

Fig 3 is the diagram that shows the electric field orientations of orthogonally polarized waves ($+45^{\circ }$ and $-45^{\circ }$) used in MIMO communication. It sets the foundation for analyzing polarization-dependent loss and cross-polarization interference caused by propagation through anisotropic vegetative media.

When considering cross polarized waves, the difference in phase variation seen by each polarization component will result in a crosstalk between the two channels carried on each polarization. XPD is the ratio of the signal (desired) on the desired polarization to the signal (undesired) transferred from the opposite polarization. This crosstalk represented as a cross-polarization discrimination XPD factor can reach 20 dB which is of great effect on communication link performance in this environment. This high level of XPD resulting in vegetative environment will limit the applicability of 6G frequencies in tropical countries where XPD can reach values of 100 times (20 dB). This means that the undesired signal from the cross polarized channel is 100 times greater than the desired signal from the channel carried on the original polarization. This indicates that energy has transferred almost from one polarization to the other polarization in both directions.

In what follows we will show the dependence of this XPD factor on medium characteristics, and we will explain how these high values of XPD arise rendering the system nonoperational and inefficient under certain circumstances and then go through the solution for this challenge by adjusting UAV location with respect to given users’ locations.

**Fig 4:**
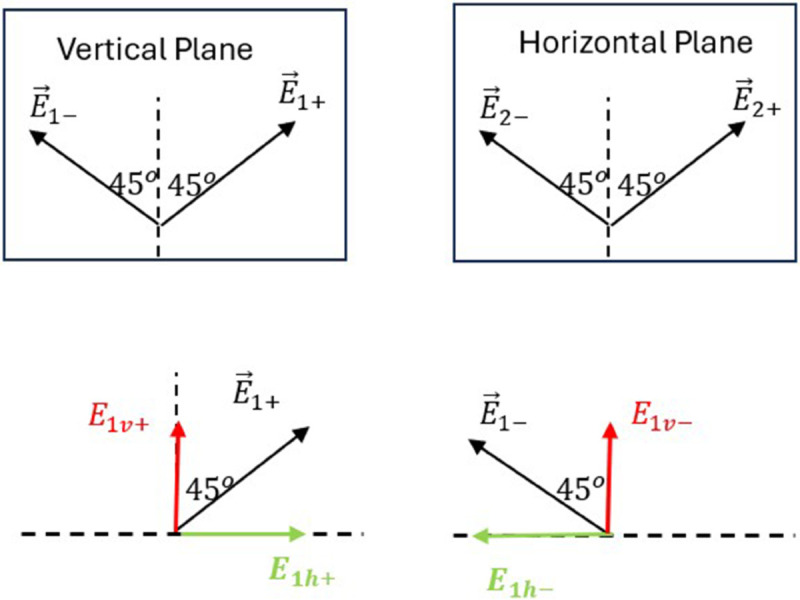
The orthogonally polarized waves in the tilted plane are first decomposed into vertical and horizontal planes. The vertical plane components are then further decomposed into vertical and horizontal directions.

First, we define the main planes and directions utilized in the following analysis and shown in Fig 4 as follows:

1. Plane of the Page: This plane contains the line of sight from the UAV to the mobile unit, and is perpendicular to the ground surface.
2. Vertical Plane: This plane is perpendicular to both the plane of the page and the ground surface.
3. Horizontal Plane: This plane is parallel to the ground surface and perpendicular to the plane of the page.
4. The tilted plane: is the plane perpendicular to the line of sight connecting the MIMO antenna on the UAV to the mobile unit on the ground. This tilted plane makes an angle *θ* with the vertical plane. This tilted plane contains the two main orthogonal polarization vectors, ${\vec{E}}_{+}$, and ${\vec{E}}_{-}$, which are oriented at  + 45^*o*^ and –45^*o*^, respectively, relative to the line lying in the plane of the page, and perpendicular to the line of sight (shown as dotted line in Fig 3).
5. Vertical Direction: It lies in the vertical plane and is perpendicular to the ground surface. This line is the intersection of the vertical plane and the plane of the page.
6. Horizontal Direction: It lies in the vertical plane and is parallel to the ground surface.

The physical antenna realization on the UAV consists of two cross-polarized patch antennas, each oriented at $\pm 45^{\circ }$ in the tilted plane.

We start by projecting the main polarization components ${\vec{E}}_{+}$, and ${\vec{E}}_{-}$ on the vertical and horizontal planes as shown in Fig 4 we get the following components:

$$E_{1+}=E_{+}\cos\theta ,\;E_{1-}=E_{-}\cos\theta \;E_{2+}=E_{+}\sin\theta ,\;E_{2-}=E_{-}\sin\theta$$ (11)

Where *θ*, as described before, is the elevation angle from the UAV to the mobile unit.

Electric field components in the vertical plane *E*_1+_, and *E*_1−_ are transmitted through vegetation. To evaluate electric field components $E_{1+}^{\prime }$, and $E_{1-}^{\prime }$ after passing through this medium, three steps are needed. The first step is to rotate axes clockwise 45^*o*^, then multiply by horizontal and vertical transmission coefficients *T*_*h*_,and $T_{v}$ respectively, finally rotate axes by 45^*o*^ counterclockwise. These steps are expressed by the following matrix equation,

$$\left[\begin{array}{c} E_{1+}^{\prime }\\ E_{1-}^{\prime } \end{array}]=[\begin{array}{cc} \cos45 & -\sin45\\ \sin45 & \cos45 \end{array}][\begin{array}{cc} T_{h} & 0\\ 0 & T_{v} \end{array}][\begin{array}{cc} \cos45 & \sin45\\ -\sin45 & \cos45 \end{array}][\begin{array}{c} E_{1+}\\ E_{1-} \end{array}\right]$$ (12)

The horizontal and vertical transmission coefficients through this vegetative medium are given by,

$$T_{h}=e^{-{\text{Loss}}_{h}/2}\cdot e^{-j\Phi_{h}}\;T_{v}=e^{-{\text{Loss}}_{v}/2}\cdot e^{-j\Phi_{v}}$$ (13)

Where *Loss*_*h*_, $Loss_{v}$, $\Phi_{h}$, and $\Phi_{v}$ are given as,

$${\text{Loss}}_{h,v}=A_{h,v}\cdot \frac{h_{V}}{\sin\theta }{\left(\frac{\lambda \sin\theta }{4\pi h_{\text{UAV}}}\right)}^{2}G_{t}\cos^{n}\left|\theta_{\max}-\theta \right|$$ (14)

$$\Phi_{h,v}=\sqrt{\varepsilon_{h,v}^{\prime }}\cdot \frac{2\pi }{\lambda }\cdot \frac{h_{V}}{\sin\theta }$$ (15)

Where $A_{h,v}$ are the vegetation loss parameters for both polarizations described by Eq (1). $h_{v}$ is the average trees height, $h_{UAV}$ is the UAV height, and *G*_*t*_ is the gain of the transmitting antenna on board of the UAV.

Electric field components in the horizontal plane *E*_2+_, and *E*_2−_are transmitted through vegetation without any relative change. To evaluate electric field components $E_{2+}^{\prime }$ and $E_{2-}^{\prime }$ after passing through the vegetative medium, we multiply both field components by *T*_*h*_ hence these components have no effect on crosstalk between MIMO channels. Crosstalk between data transmitted on *E*_+_ and that transmitted on *E*_−_ arises because of electric field components contained in vertical plane as shown by Eq (A6) which can be rewritten as,

$$E_{1+}^{\prime }=\frac{1}{2}\left[E_{1+}(T_{h}+T_{v})+E_{1-}(T_{h}-T_{v})\right]\;E_{1-}^{\prime }=\frac{1}{2}\left[E_{1+}(T_{h}-T_{v})+E_{1-}(T_{h}+T_{v})\right]$$ (16)

Therefore, XPD which is the ratio of the undesired signal passing from one polarization state to the desired signal in this polarization state is (when $E_{1+}=E_{1-}$),

$$\text{XPD}=\frac{\left|T_{h}-T_{v}\right|}{\left|T_{h}+T_{v}\right|}$$ (17)

## System analysis

**Fig 5:**
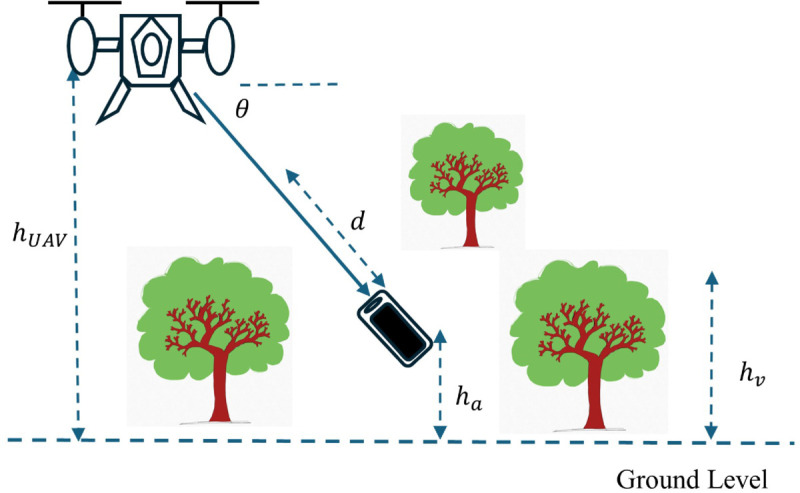
Schematic depiction of UAV-ground communication geometry and relevant spatial parameters. It illustrates vegetation depth, UAV altitude, user unit height, and elevation angle between UAV and user unit.

Fig 5 shows the schematic that outlines the spatial parameters involved in UAV–ground communication. It includes UAV altitude, user height, vegetation depth, and the elevation angle between the UAV and user. These parameters are essential for assessing signal degradation due to foliage.

The analysis consider a rescue team in a forest communicating with a UAV base station. Rescue team positions are $\left(x_{i},y_{i}\right)$, for i=1:N. The vegetation depth (travelled distance through vegetation) “d” is calculated as

$$d=\frac{\left(h_{v}-h_{a}\right)}{sin\;\theta }$$ (18)

where *θ* is the elevation angle of the path linking the UAV to a user in the forest, average mobile user height is $h_{a}\approx 1m$, average trees height is $h_{v}\approx 5m$, $h_{UAV}$ is the UAV height and is to be determined along with its location $\left(x_{UAV},y_{UAV},z_{UAV}\right)$ for optimum XPD coefficient for all the users.

The operating frequency lies in the range, 10MHz≤f≤10GHz

Consider UAV antenna beamwidth and gain using the following relation,

$$P_{\text{radiated}}=P_{0}\cos^{n}\left(\left|\theta_{\text{max}}-\theta \right|\right)$$ (19)

Where *P*_*o*_ is the radiated power at the peak of the antenna radiation pattern that occurs at $\theta_{max}$. This relation assumes isotropic radiation pattern in the azimuth and elevation directions. Beamwidths of patch antennas typically have a half-power beamwidth (HPBW) between 60^*o*^ and 120^*o*^. This HPBW can be associated with the approximate value of n, as higher n narrows the beamwidth.

- For a wider beam ($\approx 120^{o}$), n≈2.
- For a narrower beam ($\approx 60^{o}$), n≈8to10.

**Algorithm 1 Crow search algorithm for UAV XPD minimization [38–41].**

1: **Input:** Number of crows *N*_*pop*_, iterations *N*_*max*_, bounds 𝐥,𝐮 for 𝐱=[x,y,h], awareness probabilities $AP_{x},AP_{y},AP_{h}$

2: **Output:** Optimal UAV position ${𝐱}_{\text{best}}$ minimizing XPD

3: Initialize crow positions ${𝐱}_{i}\in {\mathbb{R}}^{3}$, memories ${\text{mem}}_{i}\leftarrow {𝐱}_{i}$, and fitness $f_{i}\leftarrow \text{XPD}({𝐱}_{i})$

4: ${\text{fit\_mem}}_{i}\leftarrow f_{i}$ for all $i=1,\ldots ,N_{pop}$

5: **for** *t* = 1 to *N*_*max*_ **do**

6:   **for** *i* = 1 to *N*_*pop*_ **do**

7:    Randomly select j∈{1,…,N}, j≠i

8:    **for** *d* = 1 to 3 **do** ⊳ Loop over X, Y, h

9:     **if** *d* = 1 **and** $\text{rand}>AP_{x}$ **then**

10:      $x_{i,d}^{\text{new}}\leftarrow x_{i,d}+\text{rand}\cdot ({\text{mem}}_{j,d}-x_{i,d})$

11:     **else if** *d* = 2 **and** $\text{rand}>AP_{y}$ **then**

12:      $x_{i,d}^{\text{new}}\leftarrow x_{i,d}+\text{rand}\cdot ({\text{mem}}_{j,d}-x_{i,d})$

13:     **else if** *d* = 3 **and** $\text{rand}>AP_{h}$ **then**

14:      $x_{i,d}^{\text{new}}\leftarrow x_{i,d}+\text{rand}\cdot ({\text{mem}}_{j,d}-x_{i,d})$

15:     **else**

16:      $x_{i,d}^{\text{new}}\leftarrow l_{d}+\text{rand}\cdot (u_{d}-l_{d})$

17:     **end if**

18:    **end for**

19:    Clamp $x_{i}^{\text{new}}$ within bounds [𝐥,𝐮]

20:   **end for**

21:   **for** *i* = 1 to *N* **do**

22:    Evaluate $f_{i}^{\text{new}}\leftarrow \text{XPD}(x_{i}^{\text{new}})$

23:    **if** $f_{i}^{\text{new}}<{\text{fit\_mem}}_{i}$ **then**

24:     $x_{i}\leftarrow x_{i}^{\text{new}}$

25:      ${\text{mem}}_{i}\leftarrow x_{i}^{\text{new}}$

26:      ${\text{fit\_mem}}_{i}\leftarrow f_{i}^{\text{new}}$

27:    **end if**

28:   **end for**

29: **end for**

30: ${𝐱}_{\text{best}}\leftarrow {\text{mem}}_{k}$ where $k=\arg\min_{i}{\text{fit\_mem}}_{i}$

31: **return** ${𝐱}_{\text{best}}$

### Procedure

The CSA algorithm, inspired by the intelligent behavior of crows in hiding and retrieving food, is a population-based metaheuristic that offers a robust approach to solving optimization problems. In the context of cross polarization discrimination modeling, CSA can be employed to fine-tune model parameters, improving prediction accuracy across diverse environmental conditions. By mimicking the memory and social behavior of crows, the algorithm explores and exploits the search space effectively, converging toward optimal solutions with minimal computational overhead.

The application of the crow search algorithm enables more precise estimation of signal degradation in real-world scenarios, making it a promising tool for the design and planning of high-performance wireless networks. The procedure is outlined in the following steps:

1. Calculate cross polarization discrimination XPD for all users.
2. Find the worst-case user.
3. Adjust UAV position to minimize this worst case user’ XPD.
4. Repeat until convergence.

The following summarizes the steps to calculate vegetation dielectric constant from the measured loss as a function of frequency

**Step 1: Express Loss in Terms of** $\varepsilon^{\prime \prime }(\omega )$.

The signal power attenuation due to propagation in a medium is related to the imaginary part of the refractive index $n^{\prime \prime }(\omega )$, and is given by:

$$A=\frac{4\pi n^{\prime \prime }(\omega )}{\lambda }$$ (20)

where the imaginary part of the refractive index $n^{\prime \prime }(\omega )$ is related to the dielectric constant by the relation

$$n^{\prime \prime }(\omega )=\frac{\varepsilon^{\prime \prime }(\omega )}{2n^{\prime }(\omega )}$$ (21)

this reduces the loss relation to

$$A=\frac{2\pi \varepsilon^{\prime \prime }(\omega )}{\lambda n^{\prime }(\omega )}$$ (22)

or in the form,

$$A=\frac{2\pi \varepsilon^{\prime \prime }(\omega )}{\lambda \sqrt{\varepsilon^{\prime }(\omega )}}$$ (23)

Using the above relations, we express $\varepsilon^{\prime \prime }(\omega )$ in the form,

$$\varepsilon^{\prime \prime }(\omega )=\frac{A(\omega )}{2\pi }\lambda \sqrt{\varepsilon^{\prime }(\omega )}$$ (24)

In this equation, the refractive index $n^{\prime }(\omega )=\sqrt{\varepsilon^{\prime }(\omega )}$, is initially unknown. Therefore, $\sqrt{\varepsilon^{\prime }(\omega )}$ is approximated using an average or estimated value as a starting point. An iterative algorithm is then used to refine this estimate and gradually converge to the accurate value of $\varepsilon^{\prime }(\omega )$. In practice, the procedure proceeds as follows: first, an initial estimate of the real permittivity $\varepsilon^{\prime }(\omega )$ is assumed, from which the refractive index $n^{\prime }(\omega )=\sqrt{\varepsilon^{\prime }(\omega )}$ is obtained. Using this estimate, the imaginary permittivity $\varepsilon^{\prime \prime }(\omega )$ is computed directly from the measured attenuation data through (24). Next, the value of $\varepsilon^{\prime }(\omega )$ is updated consistently with the new $\varepsilon^{\prime \prime }(\omega )$, and the process is repeated until convergence is reached.

This procedure ensures that both real and imaginary parts of the dielectric constant are physically consistent with the measured attenuation values [35]. Empirical loss data serve as the experimental basis, and the iterative refinement provides accurate frequency-dependent dielectric properties.

**Step 2: Apply the Kramers-Kronig Relation to Find** $\varepsilon^{\prime }(\omega )$.

Now that we have $\varepsilon^{\prime \prime }(\omega )$, we use the K-K integral to find $\varepsilon^{\prime }(\omega )$ using the following integral


$$\varepsilon^{\prime }(\omega )=1+\frac{2}{\pi }\int_{0}^{\infty }\frac{x\;\varepsilon^{\prime \prime }(x)}{x^{2}-\omega^{2}}\;dx$$

where $\varepsilon^{\prime \prime }(\omega )$ comes from the loss data as explained above.

## Results and discussions

This section presents the numerical simulation results derived from the dielectric modeling, XPD computation, and UAV position optimization using the CSA algorithm. The objective was to identify UAV placements that minimize XPD while accounting for frequency-dependent dielectric properties of vegetation and the spatial variability of the user distribution.

**Fig 6:**
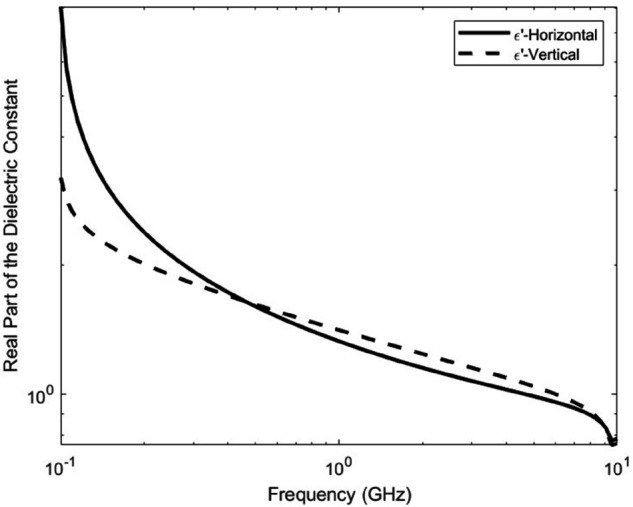
Dielectric constant (real part of $\varepsilon^{\prime }$) of vegetation for both polarizations across frequency. This figure visualizes how vegetated environments influence real permittivity, differentiating between horizontal and vertical wave interactions (*A*_1_ = 1.048 and α=0.494).

**Fig 7:**
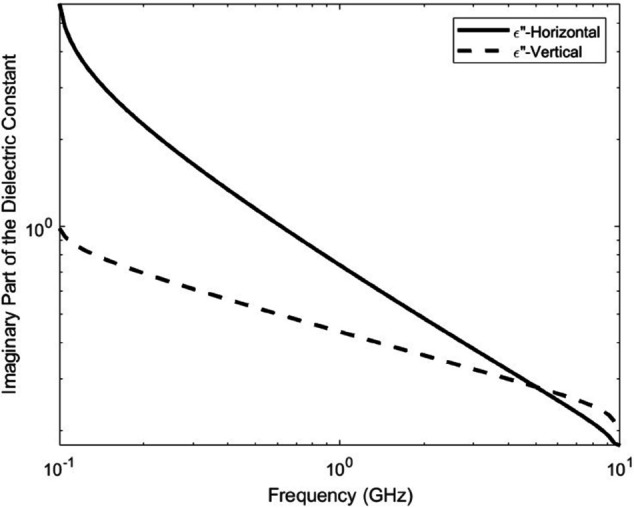
Loss factor of vegetation ($\varepsilon^{\prime \prime }$) for both polarizations across frequency. This figure visualizes how vegetated environments influence imaginary permittivity component, differentiating between horizontal and vertical wave interactions (*A*_1_ = 1.048 and α=0.494).

Figs 6 and 7 illustrate the frequency-dependent behavior of the dielectric constant and loss factor for both horizontal and vertical polarizations, corresponding to a representative forest type reported in [35]. The curves are generated using the average values of *A*_1_ and *α* given in Table 1. These plots demonstrate the anisotropic nature of vegetation media, where the real and imaginary parts of permittivity vary between polarizations across the frequency range. This dielectric behavior underpins the XPD modeling and emphasizes the need for polarization-aware UAV deployment, as conventional isotropic models would underestimate the degradation introduced by vegetation.

The dielectric constant is evaluated across a wide frequency range (100 MHz–10 GHz), as the electromagnetic response of vegetation varies strongly with frequency, and the full sweep is required to capture this behavior.

### Comparison with experimental vegetation measurements

To validate the reliability of the simulated attenuation and polarization behavior, the obtained results were compared with several well-established experimental investigations conducted in vegetated and forested environments.

The study by Meng et al. [3] systematically quantified radio wave attenuation in forests across a wide frequency range (50 MHz–2 GHz). Their measurements showed that foliage depth was the dominant factor, with losses reaching up to 32 dB greater under wet conditions than dry foliage for 1900 MHz paths and additional 17.5 dB at 240 MHz and 24.9 dB at 700 MHz under tropical rainfall. Depolarization analysis revealed 5–15 dB higher loss for vertical polarization, while seasonal variations between in-leaf and out-of-leaf conditions produced up to 8 dB difference at 462 MHz. Their findings emphasized that path loss in forested environments depends strongly on foliage depth, moisture content, polarization, and antenna height, establishing key quantitative benchmarks for future propagation modeling and forest communication system design.

The study by Zhang et al. [42] investigated 28 GHz millimeter-wave propagation in a coniferous forest using 1415 field measurements. The analysis focused on excess attenuation per unit foliage depth rather than total path loss. The authors evaluated several empirical and site-specific models. Numerically, the specific attenuation values obtained were around 6 dB/m for short vegetative paths in the ITU-R woodland model, while the Weissberger modified exponential model predicted similar rates of 2–6 dB/m depending on foliage density. In their refined site-specific models, which used LiDAR-derived foliage depth and area, the fitted attenuation coefficients were approximately 2.1–2.4 dB per meter for the initial foliage region, decreasing to 0.1 dB/m at greater depths as signal saturation occurred.

Cichoń et al. [43] investigated foliage attenuation in controlled tree-alley environments at 26–32 GHz, focusing on fixed wireless access (FWA) scenarios where the transmitter (Tx) and receiver (Rx) are aligned horizontally between consecutive trees. Field measurements were performed in two scenarios with regularly spaced London plane trees (6 m apart) and antenna heights of 2.2 m. Results showed that signal attenuation increased sharply after the second tree, marking a transition from low to high attenuation regions. The measured foliage loss exhibited an exponential growth with depth, reaching approximately 65–80 dB after 25–30 m of cumulative foliage path. When expressed per-unit foliage distance, the derived specific attenuation coefficient ranged between 1.8 dB/m and 2.5 dB/m across the 26–32 GHz band. These findings indicate that millimeter-wave signals suffer substantial attenuation—typically around 2 dB/m—when passing through dense, aligned tree canopies, with the highest loss observed for younger trees possessing denser foliage.

Cama-Pinto et al. [44] presented a broad set of measurements across VHF to microwave frequencies (50 MHz–2 GHz) to derive empirical attenuation rates for radio propagation through various forest types. Field experiments showed that specific attenuation coefficients depend strongly on frequency, foliage density, and moisture. Under typical forest conditions, the average attenuation ranged from 0.2–0.5 dB/m at VHF, increasing to 1–3 dB/m at UHF, and reaching 4–6 dB/m at 2 GHz. In wet conditions, signal loss was significantly higher—up to 32 dB greater than in dry conditions at 1.9 GHz, and roughly 17–25 dB higher at lower frequencies (240–700 MHz). The study also reported that antenna height strongly influences received power, showing an approximate 20 dB improvement when both antennas were elevated.

The attenuation and polarization-dependent behavior predicted by the proposed vegetation-aware propagation model is strongly supported by independent millimeter-wave measurement campaigns conducted under controlled foliage conditions. In particular, short-range dual-polarized experiments at 39 GHz reported in [28] demonstrate that vertically co-polarized links consistently experience lower foliage penetration loss than horizontally co-polarized links. The measurements further reveal pronounced direction-dependent attenuation and angular selectivity introduced by vegetation, as well as increased delay and angular spreads in non-line-of-sight foliage scenarios. These observations are attributed to scattering and depolarization effects caused by branches and leaves, which disproportionately affect horizontal and cross-polarized components. The same physical trends are reflected in the present model, where vegetation is treated as a stochastic attenuating and scattering medium, implicitly capturing the improved penetration efficiency and reduced depolarization associated with vertical polarization at millimeter-wave frequencies.

In addition to short-range studies, large-scale suburban macrocell measurement campaigns at 28 GHz and 39 GHz further corroborate the modeled vegetation effects. As reported in [29], extensive field measurements in vegetated suburban environments show that foliage-obstructed links exhibit significantly higher path-loss exponents and shadowing variance compared to clear line-of-sight conditions, while still allowing dominant forward-scattered components to contribute to the received signal. Importantly, these studies demonstrate that dense-leaved trees can impose excess attenuation comparable to that of building obstructions, whereas sparse vegetation permits partial transmission accompanied by increased angular dispersion. Such findings are consistent with the assumptions underlying the proposed framework, which does not treat vegetation as an opaque blocker but rather as a frequency- and geometry-dependent propagation medium. The agreement between the predicted attenuation trends and experimentally derived path-loss behavior across different vegetation densities supports the applicability of the proposed model to realistic suburban and campus-scale millimeter-wave deployments.

Ma et al. [45] comprehensively analyzed 95 studies (1960–2023) on electromagnetic wave propagation in vegetated environments. It classified attenuation research into empirical (67%), hybrid (21%), and equivalent (12%) model categories. Quantitatively, the review summarized that specific attenuation coefficients in forest environments typically range between 0.2 and 6 dB/m, depending on frequency, foliage type, and canopy density. At VHF (30–300 MHz), attenuation is usually 0.2–0.5 dB/m; at UHF (300 MHz–3 GHz), it increases to about 1–3 dB/m; and at SHF (3–30 GHz) and above, it can reach 4–6 dB/m, with localized cases up to 8 dB/m under dense or wet foliage conditions. Empirical models reviewed—such as Weissberger, ITU-R P.833, COST 235, and FITU-R—showed that wet tropical forests can impose 20–30 dB higher loss than dry conditions. The paper also compared advanced models (e.g., Azevedo’s, Anderson’s, and Oestges’), noting that modern measurements at 1–5 GHz yield attenuation constants around 2–3 dB/m, while millimeter-wave results (26–60 GHz) report 2–6 dB/m per unit foliage depth.

Overall, the attenuation levels and polarization-dependent behavior predicted by the proposed dielectric-based vegetation model are in strong agreement with the experimentally reported results summarized in Table 3. Across the considered frequency range, the simulated specific attenuation values fall within the same bounds reported by large-scale field measurements and controlled experiments, namely from sub-dB/m at VHF to several dB/m at UHF, SHF, and millimeter-wave bands. Moreover, the modeled sensitivity to foliage density, moisture content, and polarization is consistent with the depolarization trends and polarization imbalance observed experimentally in forested and greenhouse environments. This close correspondence confirms that the dielectric parameters and propagation assumptions adopted in this manuscript faithfully capture the dominant physical mechanisms governing wave interaction with vegetation. Consequently, the proposed model provides a physically grounded and experimentally validated basis for analyzing vegetation-induced attenuation and polarization effects, supporting its suitability for UAV-based communication system design in vegetated environments.

**Table 3:** Summary of key findings from major vegetation propagation studies.

Reference	Frequency Band	Scenario	Main Findings
[3]	VHF–UHF (50 MHz–2 GHz)	Mixed forests	Reported frequency- and moisture-dependent attenuation: about 0.2–0.5 dB/m at VHF, 1–3 dB/m at UHF, and up to 6 dB/m at 2 GHz. Wet foliage caused roughly 32 dB higher total loss under tropical conditions, with 5–15 dB polarization difference and about 8 dB seasonal variation.
[42]	28 GHz	Coniferous forest	Measured 2–6 dB/m attenuation using 1415 samples and LiDAR-derived foliage depth. Site-specific models achieved RMSE ≈ 19 dB and showed nearly linear loss growth with foliage depth up to about 20 m before saturation.
[43]	26.5–29.5 GHz	Alley trees	Observed exponential loss increase with cumulative foliage depth, reaching 65–80 dB after 25–30 m. Derived specific attenuation of 1.8–2.5 dB/m across the band, dominated by scattering in dense leaf canopies.
[44]	2.4 GHz ISM band	Greenhouse vegetation	Found attenuation of 1–2 dB/m and confirmed dependence on leaf density and water content under controlled humidity, consistent with dielectric absorption effects.
[28]	39 GHz	Dense tree foliage	Conducted dual-polarized directional measurements showing lower penetration loss for vertical co-polarization compared to horizontal. Observed increased delay and angular spreads under foliage blockage, with strong direction-dependent attenuation and depolarization effects caused by branches and leaves.
[29]	28 & 39 GHz	Suburban macrocell (vegetated)	Large-scale field measurements showed higher path-loss exponents and shadowing variance for foliage-obstructed links compared to line-of-sight. Dense-leaved trees caused excess loss comparable to building blockage, while sparse vegetation enabled forward-scattered components with increased angular dispersion.
[45]	30 MHz–100 GHz (VHF–mmWave)	Review	Analyzed 95 studies (1960–2023) showing typical foliage attenuation of 0.2–6 dB/m depending on frequency, canopy density, and moisture. Identified hybrid empirical–simulation models as most accurate for vegetation-loss prediction.

### XPD optimization procedure

**Fig 8:**
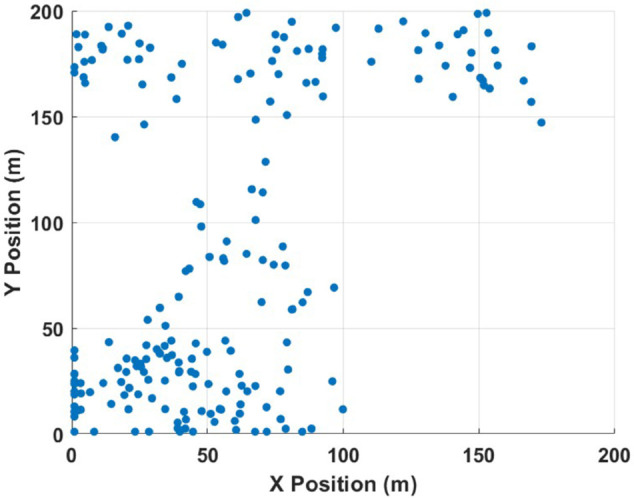
Spatial distribution of user positions (X,Y) within the designated area covered by the UAV communication link.

The scenario under study considers 200 users randomly distributed within a 200m×200m area, as illustrated in Fig 8. These users are served by a UAV acting as an aerial base station. The user locations are randomly generated in the two-dimensional (X–Y) plane to simulate realistic deployment conditions, such as emergency response teams or IoT devices dispersed in a forested environment.

To assess the impact of frequency on signal quality, we evaluate the Cross-Polarization Discrimination (XPD) for a representative user positioned at an elevation angle of 30^*o*^ relative to the UAV, which is maintained at a constant height of $h_{UAV}=200\;m$. For this user, the XPD is computed across a wide frequency range, from 100 MHz to 10 GHz.

**Fig 9:**
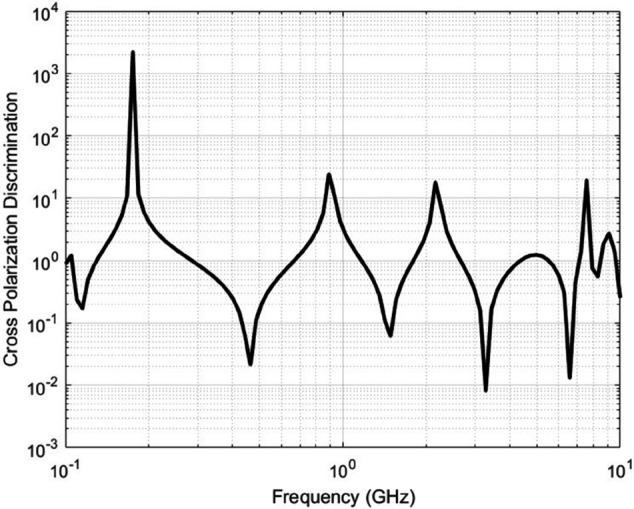
Cross Polarization Discrimination as a function of frequency for the dielectric properties defined by Figs 6 and 7. A user is selected whose angle of elevation with the UAV is $\theta =30^{o}$ and the UAV height is 200 m.

Fig 9 shows the dependence of XPD factor for the following parameters:

1. Average mobile user height is $h_{a}\approx 1m$
2. Average trees height is $h_{v}\approx 5m$
3. UAV height $h_{UAV}$ = 200 m.
4. Transmitting Antenna Gain *G*_*t*_ = 6.
5. The major lobe of the transmitting antenna has an elevation angle $\theta_{max}=45^{o}$.
6. The transmitting antenna beam width is determined by factor *n* = 2

To quantify the value of XPD reached under these conditions, a user in the targeted area is selected where the angle of elevation between this user and the UAV is $\theta =30^{o}$ and the UAV height is maintained at 200 m. Frequency varies between 100 MHz and 10 GHz. Fig 9 reveals that the XPD can reach values as high as 2000, severely degrading the quality of the communication link between the UAV and the user. Such high XPD values lead to significant polarization mismatch, resulting in low signal-to-noise ratio (SNR) and rendering the MIMO system effectively non-operational. The figure shows that XPD ranges from as low as 0.005 up to 2000, underscoring the system’s sensitivity to polarization effects. These findings highlight the critical importance of optimizing the UAV’s position relative to user locations to maintain reliable and efficient communication service.

It should be emphasized that the frequency range adopted in this study (100 MHz–10 GHz) does not imply that a practical communication system would operate across the entire band simultaneously. Instead, the purpose of this wideband analysis is threefold. First, it allows us to capture the strongly frequency-dependent nature of vegetation attenuation, phase shift, and depolarization. Second, it provides generality so that the same framework can be applied to any specific narrowband system of interest (e.g., sub-GHz IoT, LTE, or mmWave 5G/6G). Third, it is consistent with ITU-R P.833-8 recommendations [35], which validate vegetation loss models across decades of frequency. By covering the entire band, our results remain relevant to a broad spectrum of UAV communication scenarios, while still being directly applicable to narrowband deployments.

**Fig 10:**
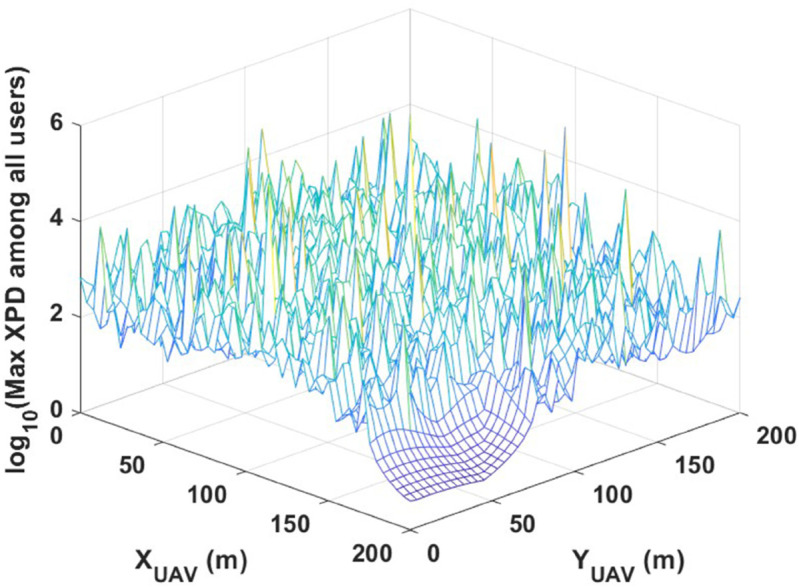
Maximum value of XPD of all users is recorded while UAV lateral position varies keeping its height at 200 m.

To evaluate the sensitivity of the system’s XPD to variations in its parameters, we first analyzed the effect of changing the UAV’s lateral position while keeping its altitude fixed at $h_{UAV}=200m.$ Fig 10 illustrates the worst-case XPD values as a function of UAV lateral position. The figure clearly shows that, without optimization, XPD can reach excessively high values. This highlights the importance of optimizing UAV positioning to minimize polarization interference and enhance system performance.

**Fig 11:**
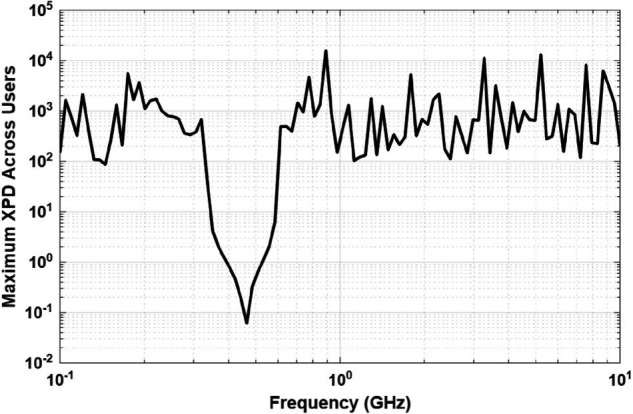
Variation of Cross-Polarization Discrimination (XPD) with elevation angle for a fixed UAV height of 200 m. Elevation angles are varied from 20^*o*^ to 89^*o*^, and the maximum XPD values are computed across different operating frequencies. The figure highlights how XPD behavior changes with elevation angle.

Fig 11 explores how XPD varies with the elevation angle between the UAV and users. In this analysis, the UAV altitude is fixed at 200 m, and the elevation angle is varied between 20^*o*^ and 89^*o*^. At each operating frequency, we calculate the maximum XPD that occur in this range of elevation angles. The figure shows that XPD remains highly sensitive to elevation angle and this emphasizes the need to consider geometric factors in system design and optimization.

**Fig 12:**
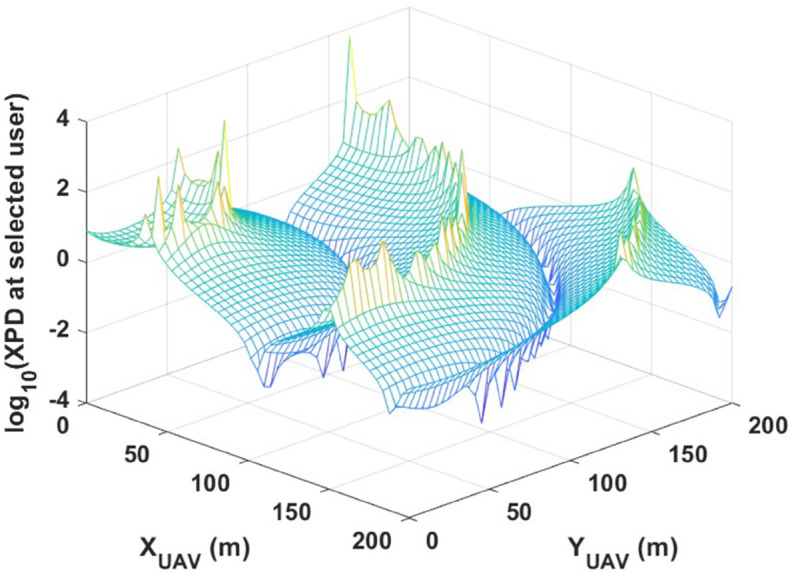
A user is selected and its XPD value is calculated while UAV lateral position varies keeping its altitude at $h_{UAV}=200m$.

Fig 12 further supports system sensitivity to varying its parameters by tracking the XPD value for a single selected user. Here again, the UAV altitude is fixed at $h_{UAV}=200\;m$ while its lateral position varies. The resulting XPD values for the selected user also demonstrate high sensitivity, confirming that individual users suffers significant signal degradation in the absence of proper UAV placement.

**Fig 13:**
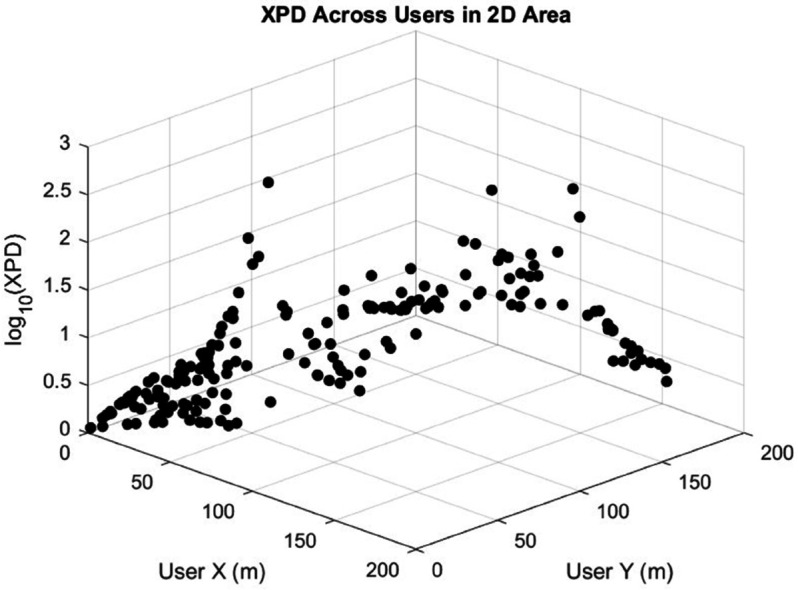
XPD values for all users without applying the optimization algorithm, with UAV positioned at $X_{UAV}=100\;m$, $Y_{UAV}=100\;m$, and $h_{UAV}=200\;m$. The figure illustrates that, in the absence of optimization, some users experience extremely high XPD values, leading to severe interference between MIMO channels and potential information loss.

In Fig 13, we show the XPD values for all users when the UAV is placed at a fixed position $\left(X_{UAV},Y_{UAV},h_{UAV})=(100\;m,100\;m,200\;m\right)$ without applying any optimization algorithm. The results reveal that several users experience extremely high XPD values, indicating severe polarization crosstalk. In such cases, the information transmitted on one MIMO channel can be almost entirely obstructed by interference from the other channel, significantly degrading system performance.

#### XPD oOptimization results.

**Fig 14:**
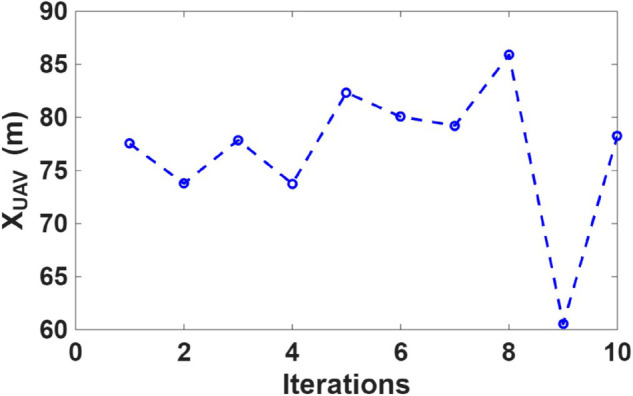
Optimal X-coordinate of the UAV position.

**Fig 15:**
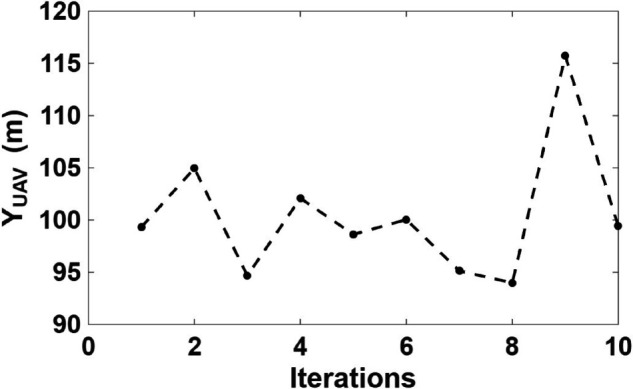
Optimal Y-coordinate of the UAV position.

**Fig 16:**
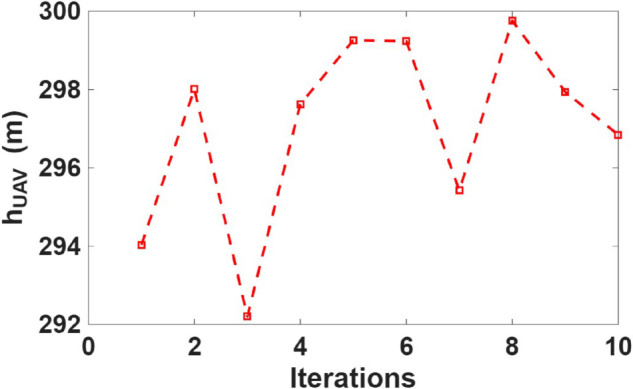
Optimal UAV altitude derived from the optimization algorithm.

Figs 14, 15, and 16 show the optimal UAV position in the X, Y, and Z directions, respectively, obtained using CSA optimization algorithm. The UAV altitude converged around 300 m, ensuring optimal link quality and vegetation-induced depolarization effects. The lateral (X, Y) positions and UAV height optimized based on minimizing the maximum XPD of the worst case user across all users.

**Fig 17:**
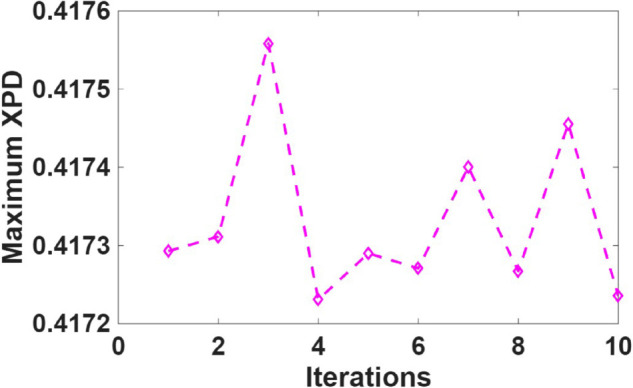
Maximum cross-polarization discrimination (XPD) observed for the worst-case user location within the coverage area.

Fig 17 presents the maximum XPD encountered by the worst-case user location. The XPD values exhibit spatial non-monotonicity, reflecting the complex interaction of electromagnetic waves with vegetative media. In contrast to traditional path loss metrics, XPD surfaces contain multiple local minima, which justifies the use of a global optimization technique such as CSA.

**Fig 18:**
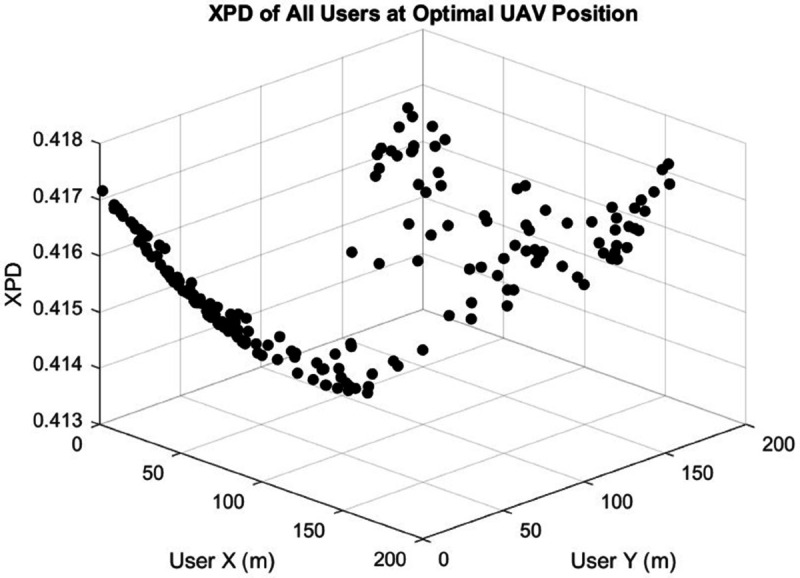
XPD values for all users after applying the optimization algorithm to determine the optimal UAV position. Compared to Fig 13, XPD values are significantly reduced and remain below 0.42 for all users, ensuring minimal inter-channel interference and a satisfactory Quality of Service (QoS) in the MIMO system.

The CSA efficiently identified optimal UAV positions that minimize the maximum XPD value, achieving a worst-case XPD of approximately 0.4. The algorithm converges in almost 5 seconds, enabling rapid UAV positioning once user location data is available. Fig 18 presents the XPD values for all users when the UAV position is optimized specifically to minimize overall XPD. The optimization results in XPD values consistently remaining below 0.42 across all users, demonstrating a substantial improvement. This optimized placement ensures a more balanced polarization environment, greatly enhancing the quality of service (QoS).

### Runtime analysis and real-time applicability

The computational cost of the proposed Crow Search Algorithm-based deployment strategy is primarily determined by the number of candidate (crow) population size $N_{pop}$, the maximum number of iterations $N_{\max}$, and the number of users $N_{u}$. The overall complexity scales as

$$𝒪\;\left(N_{pop}I_{\max}N_{u}\right),$$ (25)

Since the fitness evaluation at each candidate position involves computing the XPD values for all users.

To assess the practical feasibility of the algorithm, we measured the execution time of the CSA algorithm for the representative scenario considered in this work ($N_{u}=200$ users) using the same parameters as in Section “XPD Optimization” (population size, search bounds, and stopping criteria). The average runtime over 100 independent runs was around 5 seconds. These values are significantly lower than the time constants associated with the evolution of the environment in typical emergency-response scenarios in forests, where users are either stationary or move slowly and the vegetation is almost static. Based on the users’ movement speed within the targeted area, the system dynamically adjusts the UAV position at intervals as short as 10 seconds.

In practice, the UAV trajectory planner does not require continuous re-optimization at millisecond scales. Instead, the position can be updated every 1 minute, for example when a significant change in user distribution or link quality is detected. Given the measured runtimes, CSA can be executed well within such update intervals, leaving additional margin for communication and control tasks on the onboard computer. We therefore conclude that the proposed polarization-aware optimization is compatible with near real-time UAV deployment in the considered forested emergency communication scenarios.

#### Comparison with other search algorithms.

**Fig 19:**
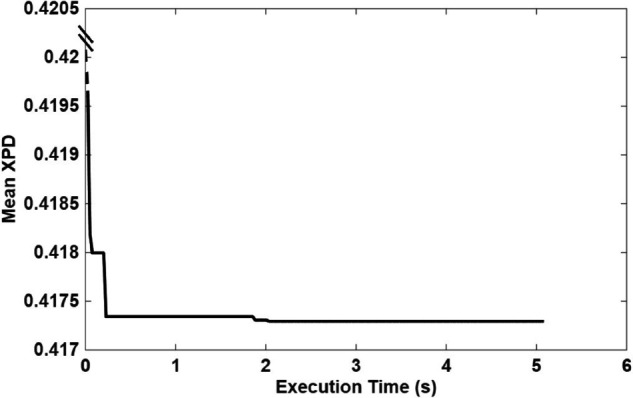
CSA runtime and convergence profile.

**Fig 20:**
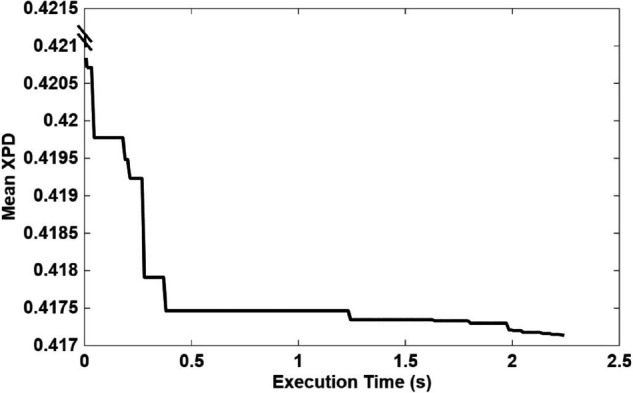
QPSO runtime and convergence profile.

**Fig 21:**
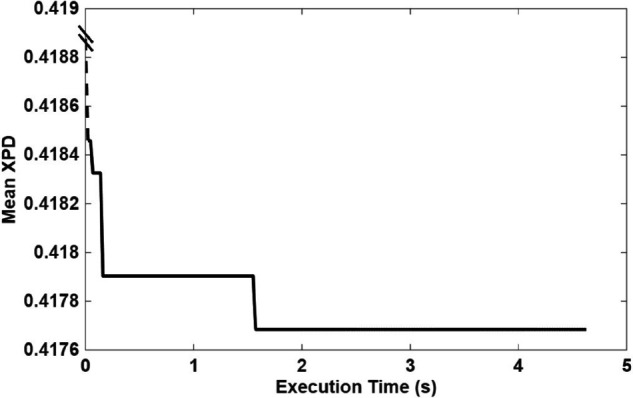
Random search runtime and convergence profile.

As illustrated in Figs 19, 20, and 21, the runtime profiles of the Crow Search Algorithm (CSA), Quantum-behaved Particle Swarm Optimization (QPSO), and Random Search are shown. Each algorithm required a comparable computational effort per iteration and converged within a similar total execution time. This similarity is expected since all three optimizers were configured with the same population size, iteration count, and fitness evaluation budget per run.

Although the three algorithms employ distinct exploration–exploitation strategies, they consistently converged to nearly identical optimal XPD values and corresponding UAV position coordinates. Despite the presence of multiple local minima across the UAV search space, all algorithms successfully located the same global minimum with comparable accuracy and convergence time. However, the CSA and QPSO algorithms demonstrated slightly higher precision than the Random Search baseline. A closer examination of the CSA convergence curve (Fig 19) reveals that it reached its near-optimal mean XPD value after approximately one-third of the total execution time, highlighting its efficiency and suitability as a lightweight yet effective alternative to QPSO.

To quantitatively reinforce this observation, Table 4 summarizes the comparative results, including average runtime, mean XPD values, and the optimal UAV coordinates $\left(x_{UAV},y_{UAV},h_{UAV}\right)$ obtained after the same number of runs.

**Table 4:** Comparison of runtime and optimization performance among CSA, QPSO, and Random Search.

Algorithm	Mean XPD	Runtime (s)	$x_{UAV}$ (m)	$y_{UAV}$ (m)	$h_{UAV}$ (m)
CSA	1.497	5.1	77.5	99.3	296.7
QPSO	1.493	2.2	79.8	97.8	295.5
Random Search	1.499	4.7	76.9	97.6	296.7

### Impact of XPD on link-level performance

The previous results have shown that vegetation-induced depolarization substantially affects the cross-polarization discrimination observed at ground users. To quantify the impact of XPD on link-level performance, we relate the co-polar and cross-polar power components to the effective signal-to-interference-and-noise ratio (SINR) and the resulting bit error rate (BER).

Let $P_{co}$ and $P_{cross}$ denote the received powers in the intended polarization and in the orthogonal polarization, respectively. The XPD (in dB) is defined as

$${XPD}_{dB}=10\log_{10}\left(\frac{P_{cross}}{P_{co}}\right).$$ (26)

Assuming that the cross-polar component acts as self-interference in a single-polarization receiver, the effective SINR can be written as

$$SINR=\frac{P_{co}}{N_{0}+P_{cross}}=\frac{P_{co}/N_{0}}{1+P_{cross}/N_{0}}=\frac{{SNR}_{0}}{1+{SNR}_{0}\cdot 10^{{XPD}_{dB}/10}},$$ (27)

where ${SNR}_{0}=P_{co}/N_{0}$ is the SNR in the absence of depolarization and *N*_0_ is the noise power. Eq (27) shows that larger XPD (stronger depolarization) directly reduces the effective SINR, especially when ${SNR}_{0}$ is moderately large.

**Fig 22:**
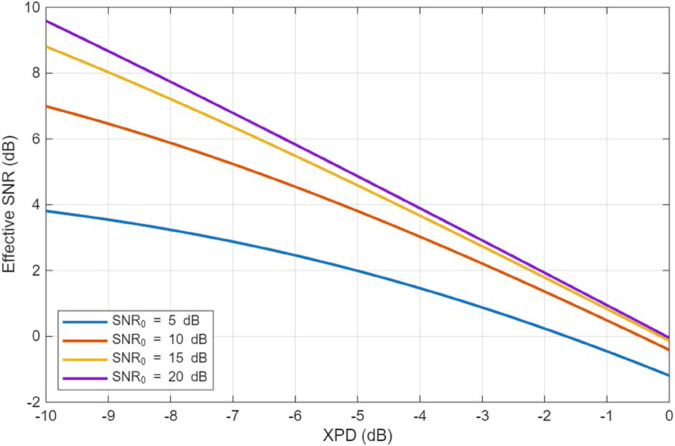
Effective SNR versus XPD.

**Fig 23:**
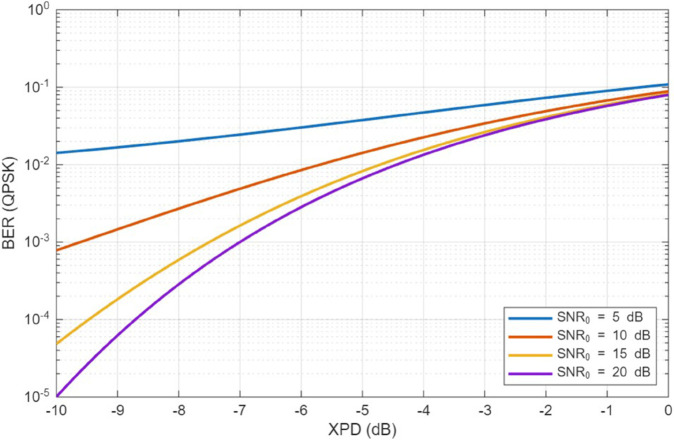
BER versus XPD (QPSK).

For a given modulation scheme, the BER can be expressed as a function of the effective SINR. For instance, for Gray-coded QPSK in an AWGN channel,

$$BER\approx Q\;\left(\sqrt{2\;SINR}\right),$$ (28)

where Q(·) is the Gaussian *Q*-function. By combining (27) and (28), we obtain a direct mapping from XPD to BER. Figs 22 and 23 illustrate example curves of effective SNR and BER versus XPD for representative values of ${SNR}_{0}$. These curves confirm that improving XPD through optimal UAV positioning can substantially reduce the BER, particularly in high-frequency bands where cross-polar leakage is pronounced.

In the context of the proposed CSA-based optimization, the XPD improvement achieved at the worst-case user translates into several dB of effective SINR gain and one to two orders of magnitude BER reduction in the illustrative QPSK scenario of Figs 22 and 23. This provides a link-level interpretation of the polarization-aware deployment strategy and demonstrates its relevance for reliable UAV-to-ground communication. The implementation of CSA optimization algorithm reduced the maximum value of XPD among all users served in the targeted area to around 0.4 (≈−4dB). The SNR in this case can be as low as 2-4 dB and BER degrades to less than 10^−2^ as shown in Figs 22 and 23. This is considered as a big achievement in improving link performance compared to the case if UAV positioning is not optimized.

## Conclusion

This study presents a comprehensive investigation into the impact of vegetation-induced polarization effects on UAV-based MIMO communication systems, emphasizing the critical role of Cross-Polarization Discrimination in determining link reliability. Unlike conventional UAV placement strategies that focus solely on minimizing path loss (The path loss variation among all users remains below 2 dB and therefore has a negligible impact on signal quality when compared with the severe degradation in SINR and BER caused by XPD). This work introduces a polarization-aware optimization framework that accounts for the complex dielectric behavior of moist foliage.

By modeling the electromagnetic properties of vegetation using the Debye relaxation and Kramers-Kronig formulations, we capture the anisotropic and frequency-dependent nature of foliage, which significantly influences signal attenuation and polarization distortion. The comparison between reported foliage attenuation measurements and the Debye-based model demonstrates that water-based dielectric modeling provides a satisfactory approximation of foliage behavior, while the present analysis itself is grounded on experimentally reported foliage attenuation data.

The analysis reveals that XPD exhibits non-monotonic spatial behavior with multiple local minima, necessitating that the algorithm searches for the global minimum among these local minima. To address this, the Crow Search Algorithm was employed to identify UAV positions that globally minimize XPD. Simulation results demonstrate that optimizing UAV placement based on XPD leads to significantly improved communication robustness, particularly in high-frequency bands where polarization crosstalk is most severe. The proposed method achieves a worst-case XPD of approximately 0.4 and converges in almost 5 seconds, making it viable for real-time deployment in emergency and disaster-response scenarios.

In conclusion, the findings underscore the importance of incorporating polarization-aware metrics into UAV deployment strategies for forested and vegetated environments. This approach not only enhances communication reliability but also paves the way for more resilient and efficient aerial network architectures in challenging terrains. It should be stressed that although UAV placement optimization was demonstrated through numerical analysis, the vegetation attenuation and dielectric parameters employed are grounded in ITU and published experimental measurements. This gives the results practical credibility and distinguishes the work from purely simulation-based studies.

## Appendix A: Derivation of cross polarization discrimination XPD factor for orthogonally polarized waves

**Fig 24:**
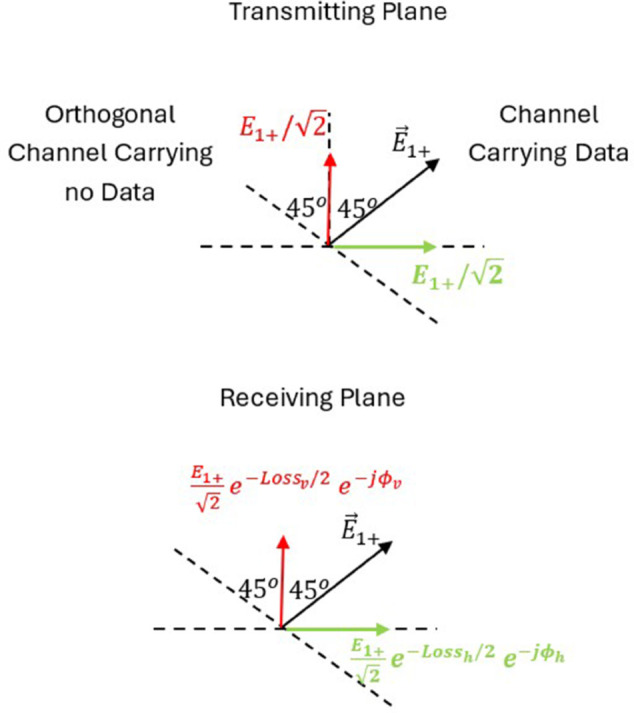
Crosstalk from $E_{1+}$ channel to $E_{1-}$ channel due to propagation in anisotropic medium.

This appendix presents the analysis for calculating cross talk between orthogonally polarized channels upon propagation in anisotropic medium. Assume data is carried on the first channel where the second channel transmits no data as shown in Fig 24. Upon propagation in this anisotropic medium composed of trees leaves and branches, the orthogonal polarization components undergo different loss and phase values as shown by Eqs (14), (15).

In this case, channel 1 (with electric field polarized  + 45^*o*^ from vertical axis) is transmitting data while channel 2 (with electric field polarized –45^*o*^ from vertical axis) is not transmitting any data.

To calculate the received field component on channel 2, the horizontal and vertical components shown in Fig 24 are projected in the direction of polarization of the idle channel yielding,


$$\frac{E_{1+}}{2}e^{-{\text{Loss}}_{v}/2}e^{-j\Phi_{v}}-\frac{E_{1+}}{2}e^{-{\text{Loss}}_{h}/2}e^{-j\Phi_{h}}$$

$$=\frac{E_{1+}}{2}\left(e^{-{\text{Loss}}_{v}/2}e^{-j\Phi_{v}}-e^{-{\text{Loss}}_{h}/2}e^{-j\Phi_{h}}\right)=R\frac{E_{1+}}{2}$$ (A1)

While the field components received by channel 1 are,


$$\frac{E_{1+}}{2}e^{-{\text{Loss}}_{v}/2}e^{-j\Phi_{v}}+\frac{E_{1+}}{2}e^{-{\text{Loss}}_{h}/2}e^{-j\Phi_{h}}$$

$$=\frac{E_{1+}}{2}\left(e^{-{\text{Loss}}_{v}/2}e^{-j\Phi_{v}}+e^{-{\text{Loss}}_{h}/2}e^{-j\Phi_{h}}\right)=T\frac{E_{1+}}{2}$$ (A2)

where ${\text{Loss}}_{h,v}$, and $\Phi_{h,v}$ are the loss (in Nepers) and phase shift of both the horizontal and vertical polarizations expressed by Eqs (14) and (15).

The second scenario when Channel 1 (with electric field polarized  + 45^*o*^ from vertical axis) is not transmitting any data while channel 2 (with electric field polarized –45^*o*^ from vertical axis) is transmitting data.

**Fig 25:**
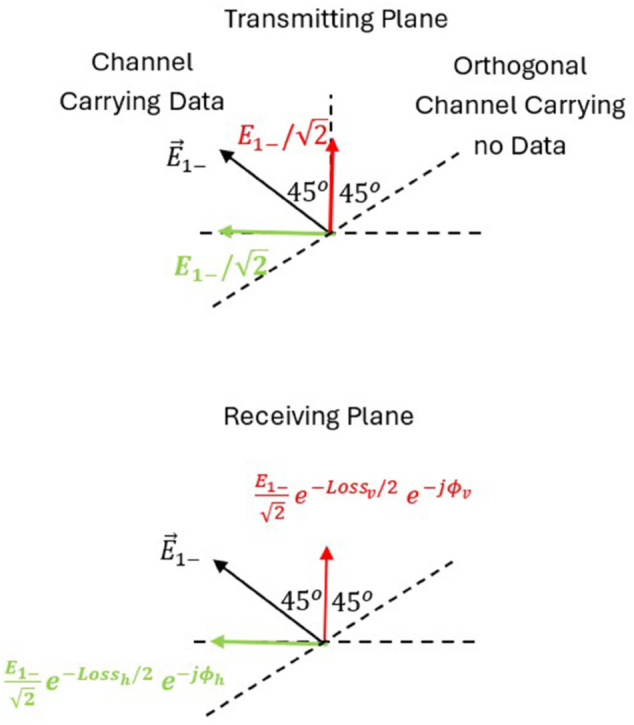
Crosstalk from $E_{1-}$ channel to $E_{1+}$ channel due to Propagation in anisotropic medium.

To calculate the received field component on channel 1, the horizontal and vertical components shown in Fig 25 are projected on the direction of polarization of the idle channel yielding,


$$\frac{E_{1-}}{2}e^{-{\text{Loss}}_{v}/2}e^{-j\Phi_{v}}-\frac{E_{1-}}{2}e^{-{\text{Loss}}_{h}/2}e^{-j\Phi_{h}}$$

$$=\frac{E_{1-}}{2}\left(e^{-{\text{Loss}}_{v}/2}e^{-j\Phi_{v}}-e^{-{\text{Loss}}_{h}/2}e^{-j\Phi_{h}}\right)=R\frac{E_{1-}}{2}$$ (A3)

Where the received signal on active channel 2 (by projecting on the direction of its polarization) will be,


$$\frac{E_{1-}}{2}e^{-{\text{Loss}}_{v}/2}e^{-j\Phi_{v}}+\frac{E_{1-}}{2}e^{-{\text{Loss}}_{h}/2}e^{-j\Phi_{h}}$$

$$=\frac{E_{1-}}{2}\left(e^{-{\text{Loss}}_{v}/2}e^{-j\Phi_{v}}+e^{-{\text{Loss}}_{h}/2}e^{-j\Phi_{h}}\right)=T\frac{E_{1-}}{2}$$ (A4)

Where *T* and *R* are given by,

$$T=e^{-{\text{Loss}}_{v}/2}e^{-j\Phi_{v}}+e^{-{\text{Loss}}_{h}/2}e^{-j\Phi_{h}}\;R=e^{-{\text{Loss}}_{v}/2}e^{-j\Phi_{v}}-e^{-{\text{Loss}}_{h}/2}e^{-j\Phi_{h}}$$ (A5)

Eqs (A1)–(A4) can be represented in a matrix form such as,

$$\left[\begin{array}{c} E_{1+}^{\prime }\\ E_{1-}^{\prime } \end{array}]=[\begin{array}{cc} T & R\\ R & T \end{array}][\begin{array}{c} E_{1+}\\ E_{1-} \end{array}\right]$$ (A6)

When two channels are transmitting different data, XPD measures how much signal intended for one channel leaks into the other channel due to imperfect polarization isolation as a way to quantify this unwanted interference. To define XPD, we compare the leaked signal (signal received on a channel that was transmitted on the other channel) to the main signal (signal received on the correct channel, as intended): For Channel 1: XPD is the ratio between the magnitude of the signal received on Channel 1 but transmitted on Channel 2 (leakage from Channel 2) to the magnitude of the main signal received on Channel 1 that was transmitted on Channel 1. For Channel 2: XPD is the ratio between the magnitude of the signal received on Channel 2 but transmitted on Channel 1 (leakage from Channel 1) to the magnitude of the main signal received on Channel 2 that was transmitted on Channel 2. In both cases, the XPD takes the same mathematical form, meaning the ratio compares the magnitude of the leaked signal to the intended signal, whether you’re looking at Channel 1 or Channel 2.

$$XPD={\left|\frac{\frac{E_{1-}}{2}e^{-{\text{Loss}}_{v}/2}e^{-j\Phi_{v}}-\frac{E_{1-}}{2}e^{-{\text{Loss}}_{h}/2}e^{-j\Phi_{h}}}{\frac{E_{1+}}{2}e^{-{\text{Loss}}_{v}/2}e^{-j\Phi_{v}}+\frac{E_{1+}}{2}e^{-{\text{Loss}}_{h}/2}e^{-j\Phi_{h}}}\right|}^{2}$$ (A7)

If signal level transmitted on both channels is equal, i.e. $E_{1+}=E_{1-}$, then XPD takes the form,

$$XPD={\left|\frac{e^{-{\text{Loss}}_{v}/2}e^{-j\Phi_{v}}-e^{-{\text{Loss}}_{h}/2}e^{-j\Phi_{h}}}{e^{-{\text{Loss}}_{v}/2}e^{-j\Phi_{v}}+e^{-{\text{Loss}}_{h}/2}e^{-j\Phi_{h}}}\right|}^{2}$$ (A8)

Or in dB as,

$$XPD\;(dB)=10\log_{10}{\left|\frac{e^{-{\text{Loss}}_{v}/2}e^{-j\Phi_{v}}-e^{-{\text{Loss}}_{h}/2}e^{-j\Phi_{h}}}{e^{-{\text{Loss}}_{v}/2}e^{-j\Phi_{v}}+e^{-{\text{Loss}}_{h}/2}e^{-j\Phi_{h}}}\right|}^{2}$$ (A9)

which is the same as described by Eqs (16) and (17).
